# Supplementary material for: MADE: A Computational Tool for Predicting Vaccine Effectiveness for the Influenza A(H3N2) Virus Adapted to Embryonated Eggs
Source: Vaccines (Basel). 2022 Jun 6;10(6):907. doi: 10.3390/vaccines10060907 (PMC9227319; doi:10.3390/vaccines10060907)
Supplement: Supplementary file 1 [file vaccines-10-00907-s001.zip › vaccines-1722947-supplementary.pdf]

Supplementary Material

Supplementary Methods

Data curation and analysis

76,489 Influenza H3N2 HA sequences from 1/1/1968 to 12/20/2018 and their associated passage histories were retrieved from the Global Initiative on Sharing All Influenza Data (GISAID). After multiple quality control (QC) steps including removing sequences with: (1) missing HA1 sequence, (2) sequences with length <=987, (3) too many ambiguous bases, (4) sequences with high divergence with other sequences (potentially due to recombination). 69,362 sequences were retained for subsequent analysis (Supplementary Table S1). Parallel to the sequence curation, we also curated passage histories of the sequences (Supplementary Table S2), and subsequently classified these sequences into different subgroups with specific passage histories including (1) egg (898 strains), (2) cell (4272 strains, a generic labeling of cell lines without referring to a specific cell type), (3) Madin–Darby Canine Kidney (MDCK) cell engineered to express increased levels of α-2,6-linked sialic acid receptors (SIAT1) (10,476 strains), (4) MDCK cell (5414 strains), (5) clinical (17,796 strains), (6) other (6961 strains) and (7) unknown (23,545 strains, no passage information is available in the database).

Sequencing alignment, phylogenetic inference, and mutational mapping

Multiple sequence alignment was performed using MUltiple Sequence Comparison by Log-Expectation (MUSCLE) with default parameter settings. Subsequently, the phylogenetic relationship (Supplementary Figure S1) and associated mutational parameters (e.g. the GTR model) were inferred using RaxML [1] Condition on the maximum likelihood estimates of the phylogenetic

relationship and the mutational parameters, the mutational mapping method is employed to infer the mutational history of the data.

The mutational mapping method [2] consists of three major steps: (1) calculate the conditional likelihood for each codon position recursively from the tip of the tree down to the root. The conditional likelihood is the probability of observing the subtree (descendant tips) conditioning on the node being A/T/C/G. This is the same calculation as the pruning algorithm. (2) sample the state of internal nodes recursively from the root of the tree down to the tips. For the root node, the probability of four bases equals to the weighted average of prior (stationary distribution) and the likelihood (i.e., conditional likelihood). For the internal nodes (non-root node), the probability of four bases equals to the weighted average of the transition probability from the ancestral node and the conditional likelihood. (3) sample the mutational history of the sequences. Conditional on the start and end base, we can simulate mutational processes from the Markov Chain and only accept those histories that are compatible with the ending base. After the mutational mapping, we effectively sampled a realization of the evolutionary history according to their posterior probability [3].

The enrichment and convergent test

Two statistical tests (the convergent and enrichment test, see maintext) were applied to identify codons driving the egg passage adaptation. After the two tests, *p*-values were calculated for each codon position and Fisher's method was used to combine these two p-values for each codon position. The combined *p*-value was then adjusted by controlling for the false discovery rate across 329 codon positions. Since the mutational mapping is a stochastic sampling method, we calculated an adjust p-value for each codon position by taking the mean of the adjusted *p*-values across 100

independent replicates. Subsequently, we extracted 17 codons based on criteria where the mean adjusted p-value <= 0.01, indicating significant adaptive evolution in the embryonated egg (Figure 1. Supplementary Figure S2). These 17 codon sites on each sequence sample are 137, 138, 145, 156, 158, 159, 160, 183, 186, 190, 193, 194, 203, 219, 225, 226 and 246.

The enrichment score, PCA and the adaptive distance

In order to measure the amount of egg passage adaptation, we first calculated the allelic enrichment score (ES) for all possible alleles (amino acids) at the 17 codons (Supplementary Figure S3). The allelic ES score is defined as the ratio as f_{egg}/f_{all}, where f_{egg} and f_{all} are the frequencies of the allele in the egg passaged sequences and all the sequences. For any input sequence, we can then extract the 17-dimensional ES scores at these 17 codons for all the sequences. By projecting the 17-dimensional vectors for all sequences into a PCA map, we can then calculate AD as the distance between the major cluster (sequences not passaged in eggs, subgroup 1 in Figure 1) and the target strain of interest. Large AD corresponds to strong egg passage adaptation while low AD represents weak egg passage adaption. Using historical VE data from 2010-2015, we performed linear regression between the VE and AD for the vaccine strains and found a strong negative correlation between AD and VE [4] (Supplementary Figure S4).

Passage history prediction based on the machine learning model

MADE contains a module that can predict the potential passage histories of the input sequence. We first translated all publicly available nucleotide acid sequences (69,362) into amino acid sequences, and then used one-hot encoding to convert them into binary vectors. Specifically, all sequences with known passage histories were labeled as 1 or 0 which indicates whether they have been passaged or not passaged in eggs. Along with the known passage information, the one-hot

encoding information over those 17 key codon sites was used for the downstream machine learning models. Given the imbalanced dataset, we employed the stratified random sampling strategy to maintain the relative proportion of sequences with different passage histories. The binary classification of egg and non-egg sequences relies on Random Forest [5] and XGboost [6], two popular machine learning methods (Supplementary Table S3 & S4). Random Forest is an effective and powerful ensemble learning method which introduces multiple regression decision tress by selecting random input explanatory variables or different regression depth and is quite useful in tackling with overfitting problem, while XGBoost, a type of gradient boosting tree method, can perform the multi-class classification in a quick, convenient, sparsity-aware and effective way. In our study, we randomly selected 70% public sequences as the training data and summarized the final prediction from 50 independent runs (Supplementary Table 3, Supplementary Figure S5).

In parallel, we also implemented a multi-class task learning model, which can classify non-egg sequences into four different passage groups including "Cell", "MDCK", "SIAT" and "Other". Since we don't have passage-type specific codons such as the egg passage condition (i.e. the 17 codons), we took the one-hot encoding information for all 329 codon sites as the input to construct the multi-class classification. Two machine learning methods were subsequently applied for similar prediction analyses, and the performance evaluation of these two methods were summarized in Supplementary Table 4 and Supplementary Figure S5.

References

1 Stamatakis, A. RAxML-VI-HPC: maximum likelihood-based phylogenetic analyses with thousands of taxa and mixed models. *Bioinformatics* **22**, 2688-2690, doi:10.1093/bioinformatics/btl446 (2006).

2 Nielsen, R. Mapping mutations on phylogenies. *Syst Biol* **51**, 729-739, doi:10.1080/10635150290102393 (2002).

3 Zhai, W., Slatkin, M. & Nielsen, R. Exploring variation in the d(N)/d(S) ratio among sites and lineages using mutational mappings: applications to the influenza virus. *J Mol Evol* **65**, 340-348, doi:10.1007/s00239-007-9019-7 (2007).

4 Chen, H., Alvarez, J. J. S., Ng, S. H., Nielsen, R. & Zhai, W. Passage adaptation correlates with the reduced efficacy of the influenza vaccine. *Clin Infect Dis*, doi:10.1093/cid/ciy1065 (2018).

5 Breiman, L. *Random forests*. Vol. 45 (2001).

6 Chen T, G. C. XGBoost: A Scalable Tree Boosting System. *Proceedings of the 22nd acm sigkdd international conference on knowledge discovery and data mining*, 785-794 (2016).

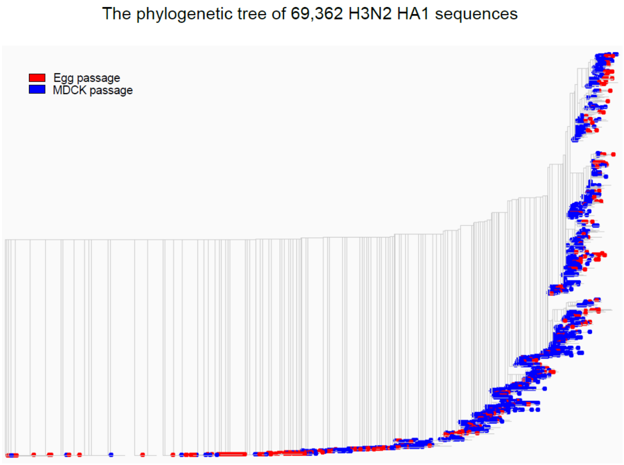

Figure S1: The maximum likelihood tree with labeled passage histories. The phylogenetic tree of the 69,362 HA1 sequences retrieved from the GISAID database. Egg and MDCK passaged isolates were labeled on the phylogenetic tree as red and blue tips, respectively.

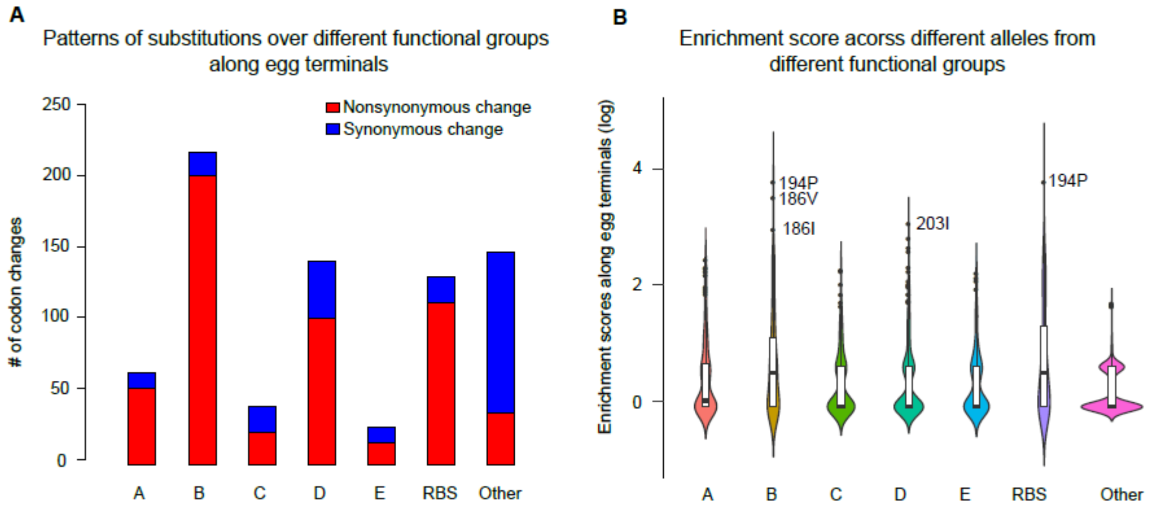

Figure S2: Patterns of egg passage adaptation. (**A**) Numbers of nonsynonymous/synonymous changes at codons from different functional groups, including five (A/B/C/D/E) antigenic surface epitope regions, receptor binding sites (RBS), as well as unclassified codon sites (Other), and (**B**) The enrichment scores across codons from different functional groups. Enrichment scores higher than 20 are labeled.

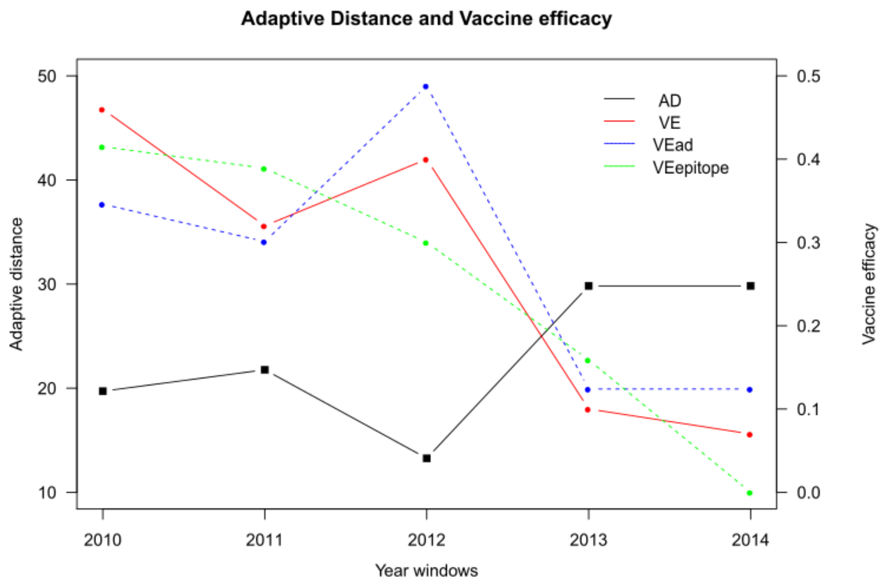

Figure S3: Correlation between the adaptive distance (AD) and vaccine efficacy (VE). The predicted vaccine effectiveness (VE_{ad}) using MADE was drawn with blue dashed line while the predicted vaccine effectiveness ($VE_{epitope}$) from changes in the dominant epitope were drawn using green dashed line [8]. The first method predicts VE based on the pattern of egg passage adaptation while the second method predicts VE based on the substitutions occurred in the antigenic epitopes.

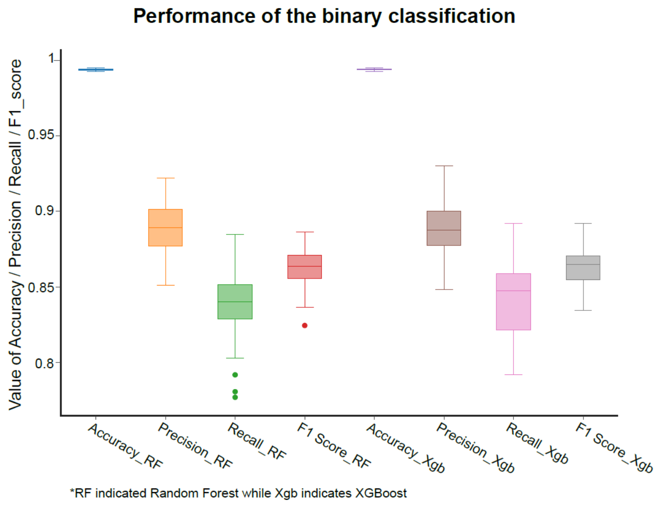

Figure S4: Performance of the binary classification. *FP: false positive, FN: false negative, Accuracy=(TP+TN)/(TP+TN+FP+FN), Precision=TP/(TP+FP). Recall=TP/(TP+FN), and F1 score=2*(precision*recall)/(precision+recall)).*

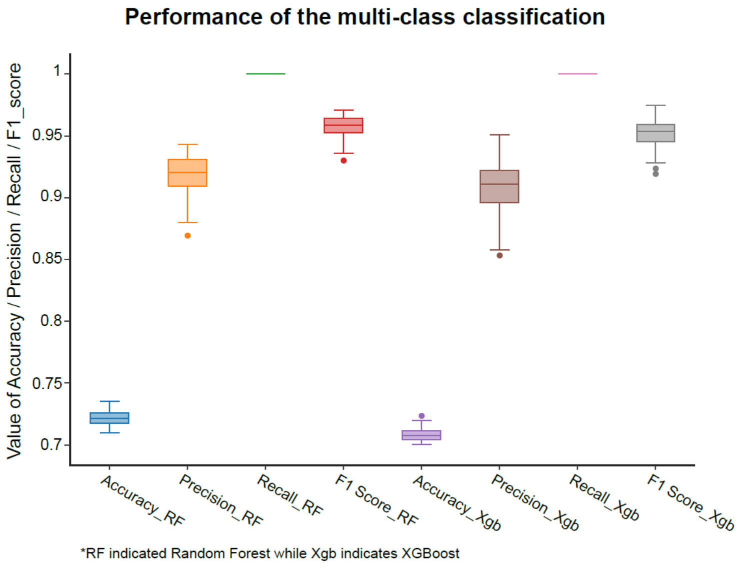

Figure S5: Performance of the multi-class classification.

Table S1: HA1 sequences collected from the GISAID database.

Please refer to the separated .xlsx file.

Table S2: Number of sequences from different passage histories.

Passage type	Egg	Cell	SIAT	MDCK	Clinical	Mix/Other	Unknown
# of strains	898	4,272	10,476	5,414	17,796	6,961	23,545

Strains labeled with multiple passage histories were classifies as "Mix/Other".

Table S3: Performance of the binary classification (Egg and non-egg).

	RF_F1	Xgb_F1
**mean**	0.8624	0.8640
**std**	0.0128	0.0124

**RF indicated Random Forest while Xgb indicates XGBoost.*
**F1=2*(precision*recall)/(precision+recall))*

Table S4: Performance of the multi-class classification (Egg, Cell, MDCK and SIAT).

	**RF_F1**	**Xgb_F1**
**mean**	0.9566	0.9521
**std**	0.0101	0.0113

**RF indicated Random Forest while Xgb indicates XGBoost.*
**F1=2*(precision*recall)/(precision+recall))*
